# High‐Frequency Mutations in *TP53* , *AXIN1* , *CTNNB1* , and *KRAS* , and Polymorphisms in *JAK1* Genes Among Mongolian HCC Patients

**DOI:** 10.1002/cnr2.70227

**Published:** 2025-05-08

**Authors:** Nomin Bold, Khurelsukh Buyanbat, Ariya Enkhtuya, Nomin Myagmar, Gerelsuren Batbayar, Zolzaya Sandag, Dolgion Damdinbazar, Nomuun Oyunbat, Tuul Boldbaatar, Ankhbayar Enkhbaatar, Gan‐Erdene Baatarjav, Taivan Nanzaddorj, Tsendsuren Oyunsuren, Gantulga Davaakhuu

**Affiliations:** ^1^ Laboratory of Molecular Biology Institute of Biology, Mongolian Academy of Sciences Ulaanbaatar Mongolia; ^2^ Department of General Surgery Second State Central Hospital Ulaanbaatar Mongolia

**Keywords:** hepatocellular carcinoma, liver cancer, mutation, p53, β‐catenin

## Abstract

**Background:**

Mongolia has the highest incidence of liver cancer worldwide, largely driven by a high prevalence of hepatitis virus infections. Mutations in oncogenes and tumor suppressor genes provide valuable insights into the molecular mechanisms of hepatocellular carcinoma (HCC).

**Aims:**

This study aimed to investigate the prevalence of mutations in key oncogenes and tumor suppressor genes in Mongolian HCC patients and to explore their molecular mechanisms, particularly in relation to hepatitis virus infections.

**Methods and Results:**

We analyzed 55 tumor tissue samples from Mongolian HCC patients (2019–2021), identifying mutations in *TP53*, *CTNNB1*, *AXIN1*, *KRAS*, and *JAK1* through sequencing. Western blotting was used to assess β‐catenin and p53 protein levels. Our findings showed p53 overexpression in tumors with *TP53* mutations (F270I and S362S), while mutations such as R213* and a short‐sequence deletion upstream of intron 7 produced premature stop codons, resulting in truncated p53 and loss of tumor suppressor function. β‐catenin accumulation was observed in tumors with *CTNNB1* mutations (D32N/Y, S33C/Y, S34V, S37P, T41A, and S45P). *CCND1* expression, a key target of the Wnt/β‐catenin pathway, was significantly upregulated in tumors harboring *CTNNB1* and *AXIN1* mutations (*p* = 0.02213). Statistical analysis revealed a positive correlation between β‐catenin and *CCND1* expression levels (*r* = 0.42703). Hepatitis virus infections were significantly associated with these mutations (*p* < 0.01), suggesting a link between viral infection and genetic alterations in HCC development. Compared to TCGA data, our cohort displayed a significantly higher mutation frequency (*p* < 0.001 and *p* < 0.05), indicating potential regional genetic and environmental influences.

**Conclusion:**

This study provides insights into the molecular mechanisms of HCC in Mongolia, highlighting distinct mutational patterns in *TP53*, *CTNNB1*, *AXIN1*, and *KRAS*. The association between hepatitis virus infections and these mutations underscores their potential oncogenic impact and may inform future therapeutic strategies for HCC in this population.

**Abbreviations**
Ala: alanine
Arg: arginine
Asp: aspartic acid
bp: base pair
*CCND1*
: *cyclin D1*
cDNA: complementary DNA
CK1α: casein kinase 1 alpha
Ct: cycle threshold
*CTNNB1*
: *catenin β‐1*
CtNorm: cycle threshold value
DNA: deoxyribonucleic acid
ECL: enhanced chemiluminescence
Gln: glutamine
Glu: glutamic acid
Gly: glycine
GSK3β: glycogen synthase kinase‐3 β
HBV: hepatitis B virus
HBx: hepatitis B viral X
HCV: hepatitis C virus
HDV: hepatitis D virus
His: histidine
HRP: horseradish peroxide
Ile: isoleucine
*JAK1*
: *Janus kinase‐1*
*KRAS*
: *Kristen rat sarcoma virus*
Lys: lysine
MAPK: mitogen‐activated protein kinase
Met: methionine
mRNA: messenger RNA
PCR: polymerase chain reaction
PRD: proline‐rich domain
Pro: proline
PVDF: polyvinylidene fluoride
qPCR: quantitative polymerase chain reaction
SAM: sterile alpha motif
Ser: serine
SNP: single nucleotide polymorphism
TAD: transcriptional activation domain
TD: tetramerization domain
Thr: threonine
*TP53*
: tumor protein 53
Val: valine
Wnt‐1: wingless and int‐1

## 1 Introduction

According to GLOBOCAN estimates, the global incidence of liver cancer reached 865 269 new cases in 2022, with a mortality‐to‐incidence ratio of 87.59% [1]. Notably, Mongolia has the highest incidence of liver cancer worldwide, with an estimated rate of 96.1 cases per 100 000 inhabitants, according to the World Cancer Research Fund International [2].

Hepatocellular carcinoma (HCC) accounts for approximately 75%–85% of all liver cancer cases [3] and is primarily associated with risk factors such as chronic hepatitis B virus (HBV) and hepatitis C virus (HCV) infections [4], as well as metabolic conditions like obesity [5]. In Mongolia, the prevalence of HBV and HCV infections remains alarmingly high, at 10% and 15%, respectively [6], significantly contributing to the country's high liver cancer burden.

At the molecular level, genomic stability is maintained through DNA repair mechanisms, in which the tumor suppressor protein p53—often referred to as the “guardian of the genome”—plays a critical role [7]. Mutations in *TP53* are among the most frequent genetic alterations in human cancers, occurring in approximately 50% of all malignancies [8]. Additionally, β‐catenin, encoded by the *CTNNB1* gene, is a key component of the cadherin complex and plays a crucial role in the Wnt signaling pathway, mediating both paracrine and autocrine signaling [9]. Mutations in *CTNNB1* often result in β‐catenin overexpression, contributing to the development of various cancers, including HCC, colorectal carcinoma, lung cancer, and ovarian cancer [10]. Given its role in maintaining cellular homeostasis, dysregulation of the Wnt/β‐catenin signaling pathway is a key driver of tumorigenesis, affecting apoptosis and cell proliferation [11].

Mutations in *AXIN1* (P312T, R398H, L445M, D545E, G700S, and R891Q) have been identified in tumors across multiple organs, including the liver, brain, lungs, and cervical spine [12]. As a key scaffold component of the β‐catenin destruction complex, *AXIN1* plays a critical role in β‐catenin regulation [13]. Overexpression of *AXIN1* leads to β‐catenin downregulation and has been linked to colon cancer [14]. Additionally, β‐catenin regulates the expression of cyclin D1, encoded by the *CCND1* gene, through the LEF‐1 transcriptional pathway [15, 16], and its accumulation in the nucleus can activate *CCND1* via the Wnt signaling pathway [17].

Kirsten rat sarcoma (*KRAS*) protein is another key regulator of cell division and growth in signal transduction pathways [18]. However, mutations in *KRAS* lead to hyperactivation of the RAS/MAPK signaling pathway, driving uncontrolled cell proliferation and contributing to the development of pancreatic [19], colorectal [20], lung [21], and liver cancers [22]. Similarly, *Janus kinase* 1 (*JAK1*) mutations have been implicated in multiple cancers, including leukemia [23], breast cancer [24], lung cancer [25], and liver cancer [26]. High‐frequency *JAK1* mutations, such as S729C, S703I, and V617F [27], contribute to tumor progression by acting as either oncogenic drivers [28] or tumor suppressors [24]. The mutational frequency of *JAK1* alterations in HCC has been reported to be 9.1% [29].

Despite extensive global research on HCC pathogenesis, there remains an urgent need to elucidate the molecular mechanisms underlying liver cancer in Mongolia. This study aims to investigate mutations in *TP53*, *CTNNB1*, *AXIN1*, *KRAS*, and *JAK1* genes and their associations with hepatitis virus infections in Mongolian HCC patients.

## 2 Materials and Methods

### 2.1 Tissue Samples

As part of the research project titled “Study of Some Gene Expressions and Their Effects on Liver Cancer Development” (conducted from 2020 to 2023 by the Laboratory of Molecular Biology, Institute of Biology, Mongolian Academy of Sciences, Ulaanbaatar, Mongolia), liver tumor and peritumoral tissue samples were collected from 55 patients diagnosed with HCC. All patients underwent surgical intervention at the Second State Central Hospital in Ulaanbaatar, Mongolia, between June 2021 and December 2022.

The study was approved by the Ethics Committee of the Ministry of Health of Mongolia on June 3, 2021 (Ethical approval no. 220; National Registration No. 202300080). Informed consent was obtained from all individual participants prior to sample collection. The tissue samples were stored at −80°C for subsequent molecular analysis.

The cohort comprised 18 patients with HCV infection, 10 patients co‐infected with HBV and hepatitis D virus (HDV), and 2 patients with triple infection (HBV, HCV, and HDV). Additionally, 10 patients had HCC unrelated to hepatitis virus infection. Overall, 81.8% of the patients had chronic hepatitis, and 91% were over 50 years of age. The gender distribution was 45% male and 55% female (Supporting Information S2).

### 2.2 Nucleic Acid Extraction and PCR Amplification

Genomic DNA and total RNA were extracted from the liver tissue samples using the QIAGEN QIAamp DNA Mini Kit (Cat. No. 51104) and the QIAGEN RNeasy Mini Kit (Cat. No. 74104), according to the manufacturer's instructions. The extracted samples were stored at −20°C for subsequent analysis. To obtain complementary DNA (cDNA), total RNA was reverse‐transcribed using SuperScript II Reverse Transcriptase (Thermo Fisher Scientific, USA) and oligo(dT) primers. Specific cDNA fragments were amplified using the polymerase chain reaction (PCR) method. The primer sequences used for PCR amplification are listed in Table 1.

**TABLE 1:** Primer sequences used in PCR amplification of *TP53*, *CTNNB1*, *KRAS*, *AXIN1*, *JAK1*, *CCND1*, and *GAPDH*.

Target genes	Primers' sequence
*TP53*	F: 5′‐AGGGCAGCTACGGTTTCCGTCT‐3′
	R: 5′‐TCAGTCTGAGTCAGGCCCTTCTGTC‐3′
*CTNNB1*	F: 5′‐ATGGCTACTCAAGCTGATTTG‐3′
	R: 5′‐CATCTAATGTCTCAGGGAACA‐3′
*KRAS*	F: 5′‐ATGACTGAATATAAACTTGTGGTAG‐3′
	R: 5′‐CCCAGATTACATTATAATGCATTTT‐3′
*AXIN1*	F: 5′‐AGAGGTGATGGTGCTGCTT‐3′
	R: 5′‐AGCCCCCTCCTCACTGAC‐3′
*JAK1*	F: 5′‐TTCTGGGACCCTGATGGATT‐3′
	R: 5′‐AAGGACATTTCTTGCTGCCA‐3′
*CCND1*	F: 5′‐TACACCGACAACTCCATCCGGC‐3′
	R: 5′‐TTGATCACTCTGGAGAGGAAGCG‐3′
*GAPDH*	F: 5′‐CTGGGCTACACTGAGCACC‐3′
	R: 5′‐AAGTGGTCGTTGAGGGCAATG‐3′

Abbreviations: F, forward primer; R, reverse primer.

### 2.3 Quantitative PCR (qPCR) for CCND1 Expression

qPCR was performed to assess the expression of the *CCND1* gene, with *GAPDH* as the reference gene. The qPCR was conducted using the Mx3000P and Mx3005P qPCR systems (Stratagene, USA) with ZAC SYBR Green 2× qPCR Premix (Cat. No. PCR003‐001). The specific primers for each gene are listed in Table 1. *CCND1* expression was quantified by comparing the cycle threshold (Ct) values. Normalized Ct values (CtNorm) were calculated, and statistical significance was assessed using a *t*‐test in Microsoft Excel. The data were visualized using box plots, generated in SigmaPlot 14.0 software. The relationship between β‐catenin overexpression and downstream signaling gene expression (*CCND1*) was evaluated by calculating the Spearman correlation coefficient (*r*) in Excel.

### 2.4 Mutational Analysis

Mutations in *TP53*, *CTNNB1*, *AXIN1*, *KRAS*, and *JAK1* genes were identified through DNA sequencing of the PCR‐amplified products. Sequencing was performed by Macrogen Inc. (Seoul, Korea). The sequence data were analyzed using SnapGene 2.3.2 software to identify mutations and polymorphisms, which were further compared to reference sequences from the NCBI database using SnapGene 2.3.2 and ApE programs.

Mutational analysis of the HCC cohort was performed with Maftools in R Studio. Single nucleotide polymorphisms (SNPs) were excluded from further analysis, and the focus was placed on the key genes *TP53*, *CTNNB1*, *AXIN1*, and *KRAS*, and 39 HCC samples were analyzed for somatic mutations using Mutation Annotation Format (MAF) files. Mutation frequencies, distributions, and mutation types (e.g., C>T and T>C) were determined. Transition/transversion (Ti/Tv) ratios were calculated to assess the nature of mutations. Multi‐hit mutations were also analyzed to identify potential hotspot regions. The findings were compared to data from the TCGA data set (*N* = 415).

Statistical analysis was performed to evaluate the effects of age and gender on mutation frequencies using an ANOVA test. The relationship between viral infections (HBV, HCV, and HDV) and mutational status was analyzed using multiple linear regression in XLSTAT (Addinsoft, USA). Associations between mutations and clinical variables were assessed using Spearman's correlation in XLSTAT. Odds ratios (OR) were calculated to compare mutation frequencies in our cohort with the TCGA data set. Differences in mutation distribution and tumor mutation burden (TMB) were analyzed using Maftools in R Studio, and statistical significance was determined using *p* values.

### 2.5 Protein Expression Analysis by Western Blotting

Total proteins were extracted from liver tissues using a tissue lysis buffer containing 1% SDS and 1 mM PMSF. The protein concentration was measured using a BCA assay (Thermo Fisher Scientific). Proteins were separated by SDS‐PAGE on 10% polyacrylamide gels and transferred to polyvinylidene fluoride (PVDF) membranes. To detect p53 and β‐catenin expression, the membranes were incubated with monoclonal antibodies: p53 (MMab, Bio SB), β‐catenin (MMab, Bio SB), and GAPDH (Santa Cruz Biotechnology) at appropriate dilutions for 2 h at room temperature. After primary antibody incubation, membranes were washed and incubated with horseradish peroxidase (HRP)‐conjugated rabbit anti‐mouse secondary antibody (Jackson ImmunoResearch, USA) for 1 h. Protein bands were visualized using an enhanced chemiluminescence (ECL) detection system (Bio‐Rad Laboratories, USA). The intensities of the protein bands were quantified using ImageJ software (NIH, USA). The expression levels of β‐catenin and p53 proteins were normalized to the corresponding GAPDH band intensity for each sample to control for loading differences. The normalized values were used to compare the relative protein expression between HCC tumor tissues and peritumoral normal tissues. The quantitative results were displayed as graphs next to the corresponding Western blot images.

## 3 Results

### 3.1 Mutations and Polymorphisms in Key Oncogenes

**FIGURE 1:**
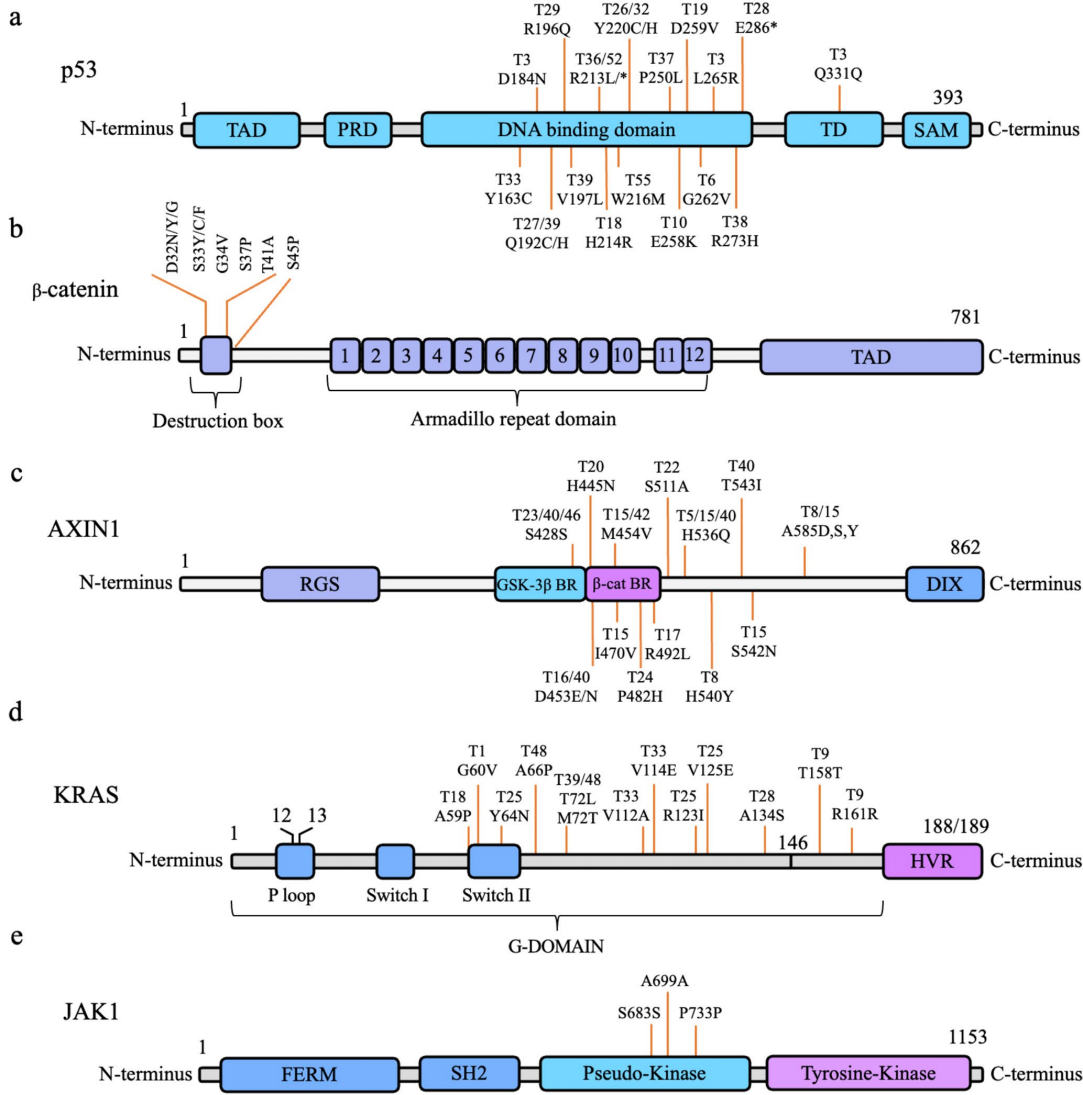
Domain structures of p53, β‐catenin, AXIN1, KRAS, and JAK1 proteins with mutations indicated by codon changes. (a) Mutations in p53 are shown within its domain structure: Transcriptional activation domain (TAD), proline‐rich domain (PRD), DNA‐binding domain (DBD), tetramerization domain (TD), and sterile alpha motif (SAM). (b) Mutations in β‐catenin are highlighted within its domain structure: Armadillo repeats (1–12) and TAD. (c) Mutations in AXIN1 are shown within its domain structure: Regulator of G protein signaling domain (RGS), GSK‐3β binding region (GSK‐3β BR), β‐catenin binding region (β‐cat BR), and disheveled‐interacting domain (DIX). (d) Mutations in KRAS are indicated within its domain structure: C‐terminal hypervariable region (HVR). (e) Mutations in JAK1 are shown within its domain structure: FERM domain (4.1 Protein, Ezrin, Radixin, and Moesin Domain), Src homology 2 domain (SH2), pseudokinase domain, and tyrosine kinase domain.

**FIGURE 2:**
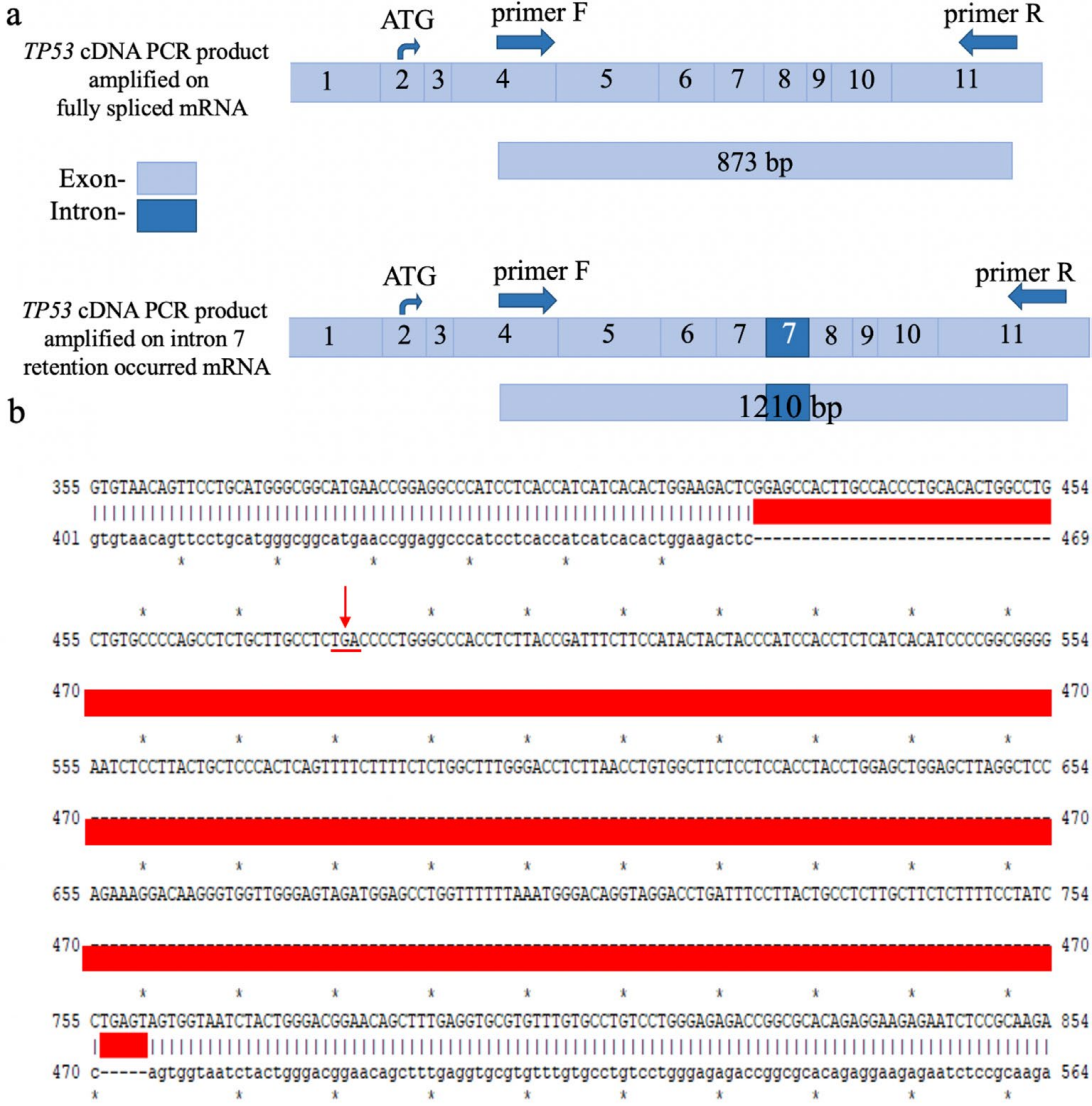
Effect of a deletion at the 5′ splice site (–CAGGTCA–) in the HCC tumor tissue sample T20. (a) Comparison of *TP53* cDNA PCR products amplified from fully spliced mRNA and mRNA with intron 7 retention in the tumor sample T20. Exons are shown in light blue, and introns in dark blue. (b) Alignment of the *TP53* cDNA sequence from sample T20 with the reference sequence. The retained intron 7 sequence is highlighted in red, and the arrow indicates a premature stop codon.

DNA sequencing of 55 HCC patient samples identified mutations and polymorphisms in the *TP53*, *CTNNB1*, *AXIN1*, *KRAS*, and *JAK1* genes (Figures S1–S5). *TP53* exons 4–11 were amplified and sequenced, revealing mutations in 23 cases (41.8%). Among these, 84% were missense mutations within the p53 DNA‐binding domain (Figure 1a). Notable amino acid substitutions included glutamic acid (Glu) to lysine (Lys) at position 258 (sample T10), glutamine (Gln) to histidine (His) at position 192 (sample T39), and valine (Val) to methionine (Met) at position 216 (sample T55) as shown in Figure 1a. Additionally, an intronic mutation in intron 7 was detected in tumor sample T20, resulting in the retention of the intron and the creation of a premature stop codon due to a deletion of the sequence (–CAGGTCA–) at the 5′ splice site (Figure 2b), as identified using ApE software.

*CTNNB1* mutations were detected in 16 cases (29.09%), all of which were heterozygous missense mutations confined to exon 3 (Figure 1b). Among these, 53% occurred at phosphorylation sites (S33, S37, T41, and S45), which are critical for regulating β‐catenin degradation. The remaining 47% were found at positions D32 and G34, essential for β‐catenin degradation via the ubiquitin‐proteasome pathway. These alterations may lead to the accumulation of stabilized β‐catenin, contributing to aberrant Wnt/β‐catenin signaling, a well‐known oncogenic pathway. *AXIN1* mutations were observed in 12 cases (21.8%), primarily localized to the β‐catenin binding domain or adjacent regions (Figure 1c). Single nucleotide polymorphisms (SNPs) were detected in six cases (10.9%), including the common S428S polymorphism in three samples. A stop codon mutation at position 464 (Glu to stop codon) was identified in samples T3 and T4, resulting in *AXIN1* inactivation. Additionally, an isoleucine (Ile) to valine (Val) substitution at position 470 was observed in sample T25, potentially disrupting the α‐helix structure of *AXIN1* while retaining its β‐catenin binding activity. This suggests that *AXIN1* may still interact with β‐catenin despite the structural change. *KRAS* mutations were detected in 10 cases (10.9%), primarily localized within the G‐domain between positions 59 and 146 (Figure 1d). These mutations impair the GTPase activity of *KRAS*, resulting in constitutive activation of the RAS/MAPK signaling pathway, a key driver of tumorigenesis. Notably, *KRAS* mutations are frequently observed in various cancers, including pancreatic, colorectal, and lung cancers, where they are associated with high mutation frequencies.

Polymorphisms in the *JAK1* gene were detected in 45.45% (*n* = 25) of the samples, all localized within the pseudokinase domain (Figure 1e). The most common polymorphisms included A699A, identified in 2 samples, S683S in 6 samples, and P733P in 17 samples. These polymorphisms are of particular interest as they may influence *JAK1*'s role in cytokine signaling, a pathway implicated in the pathogenesis of various cancers, including HCC.

### 3.2 Association of Genetic Alterations With Hepatitis Virus Infections in HCC

**FIGURE 3:**
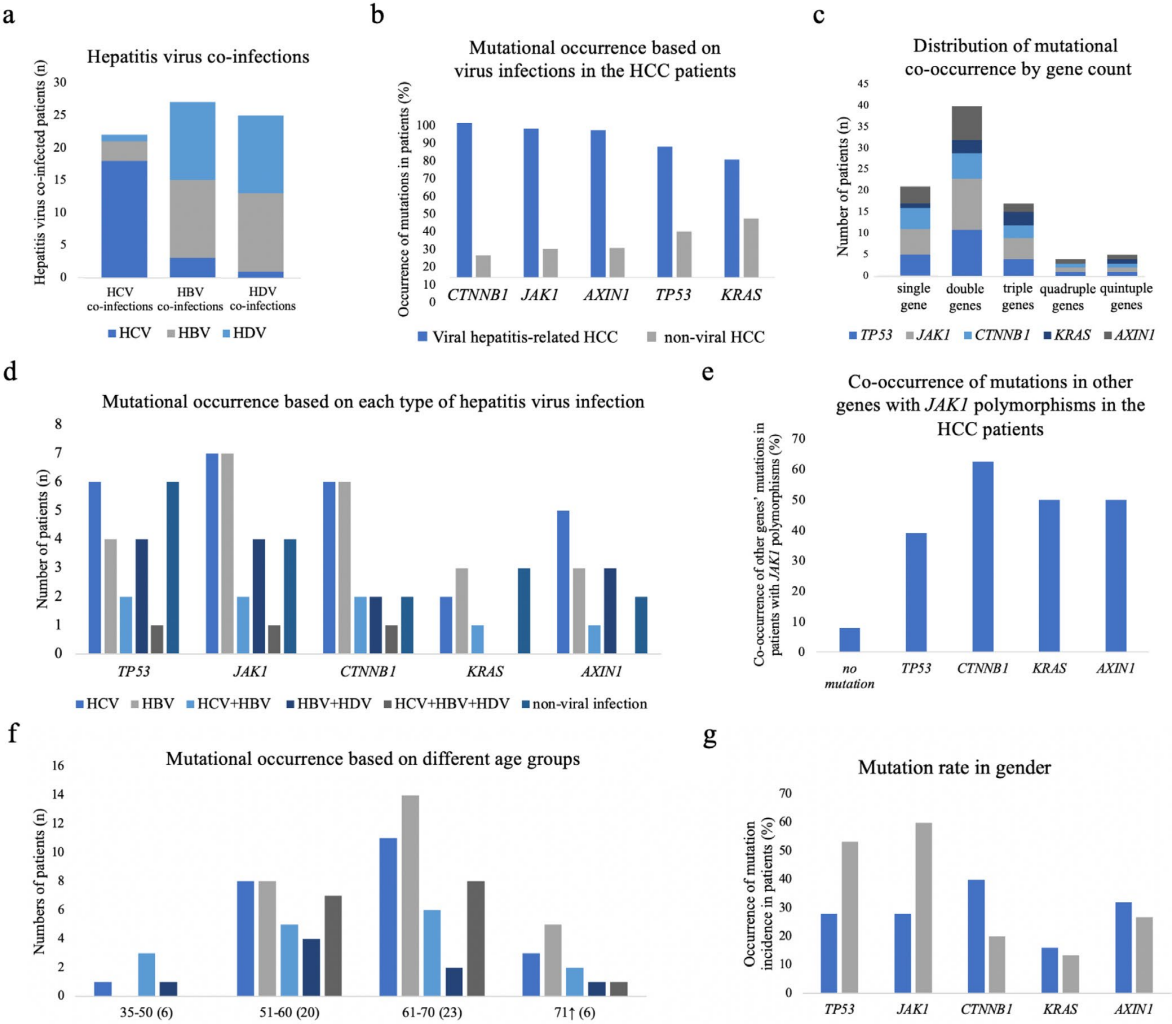
Comparison of mutations and hepatitis virus infections in HCC patients. (a) Hepatitis virus co‐infections in HCC samples. (b) Mutation frequencies of *TP53*, *CTNNB1*, *AXIN1*, *KRAS*, and *JAK1* based on hepatitis virus infections in HCC samples. (c) Co‐occurrence of mutations across different genes, categorized by the number of mutations per patient. (d) Frequency of *TP53*, *CTNNB1*, *AXIN1*, *KRAS*, and *JAK1* mutations in HCC patients with different types of hepatitis virus infections, as well as in non‐viral HCC cases. (e) Co‐occurrence of *JAK1* polymorphisms with *TP53*, *CTNNB1*, *AXIN1*, and *KRAS* mutations in HCC patients. (f) Mutation frequencies of different genes stratified by age groups. (g) Mutation frequencies of different genes stratified by gender.

In this study, 85.4% of HCC patients were infected with hepatitis viruses, with 50.9% testing positive for HBV, 40% for HCV, and 23.63% for HDV. The remaining 14.54% were classified as non‐hepatitis‐related HCC cases. Among the mono‐infected HCC patients, HCV was the most prevalent infection (77.27%), followed by HBV (42.85%). Notably, 46.42% of HBV‐related HCC cases were co‐infected with HDV, while 17.85% were co‐infected with HCV (Figure 3a).

Mutations in tumor suppressor genes (*TP53* and *AXIN1*) and oncogenes (*CTNNB1* and *KRAS*) were significantly more frequent in hepatitis virus‐related HCC cases compared to non‐viral HCC cases (Figure 3b). Statistical analyses revealed a strong association between mutation occurrence and viral infections (*p* < 0.01), as well as with specific hepatitis virus types (*p* < 0.01). Among these, *TP53* mutations were the most prevalent and exhibited the strongest correlation with hepatitis virus infections (*p* < 0.01).

Co‐occurrence of mutations was commonly observed, with single and double mutations being more frequent than quadruple or quintuple mutations (Figure 3c). In HCC cases, the frequency of mutations was significantly higher in patients with mutations in two genes compared to those with mutations in a single gene (*p* < 0.05), indicating a correlation between mutation frequency and the co‐occurrence of mutations. Additionally, among cases with single‐gene mutations, *TP53*, *CTNNB1*, and *AXIN1* mutations were the most frequently observed, whereas *KRAS* mutations were less common.

Mutation frequencies were elevated in HCC patients with HCV and/or HBV infections (Figure 3d). In HCV‐associated HCC, mutations were detected across all studied genes, with *TP53* and *CTNNB1* mutations occurring in 36% of cases, followed by *AXIN1* (27%) and *KRAS* (13%). *TP53* mutations were strongly associated with HCV infection (*p* < 0.01), while *CTNNB1* mutations showed a moderate association (*p* < 0.05).

In HBV‐related HCC cases, *CTNNB1* and *AXIN1* mutations were the most prevalent (both at 29%), with other mutations occurring less frequently. HBV and HBV + HDV co‐infections were significantly associated with *CTNNB1* and *KRAS* mutations (*p* < 0.05), whereas HBV + HDV co‐infections specifically correlated with *AXIN1* mutations (*p* < 0.05). *KRAS* mutations were the least frequent, observed in only 13% of HCV‐related and 14% of HBV‐related cases. Notably, no *KRAS* mutations were detected in HBV + HDV co‐infected cases.

*JAK1* polymorphisms were identified in 50% of both viral and non‐viral HCC cases. Notably, 76% of *JAK1* polymorphisms occurred in conjunction with mutations in other genes. The most frequent co‐occurrence was with *CTNNB1* mutations (62.5%), followed by *KRAS* and *AXIN1* mutations (both at 50%) (Figure 3e). In addition, *JAK1* polymorphisms were more prevalent in HCC patients over 50 years of age (Figure 3f), a group in which *TP53* mutations were also more common. In contrast, *CTNNB1* mutations were predominantly observed in younger patients (aged 35–50 years). While no significant gender‐based differences in mutation frequencies were found (*p* > 0.05), mutations in *TP53*, *CTNNB1*, and *JAK1* were more frequent in females (*p* < 0.01), whereas mutations in *CTNNB1*, *AXIN1*, and *KRAS* were more common in males (Figure 3g).

### 3.3 Mutational Analysis and the Distribution in the HCC Cohort

**FIGURE 4:**
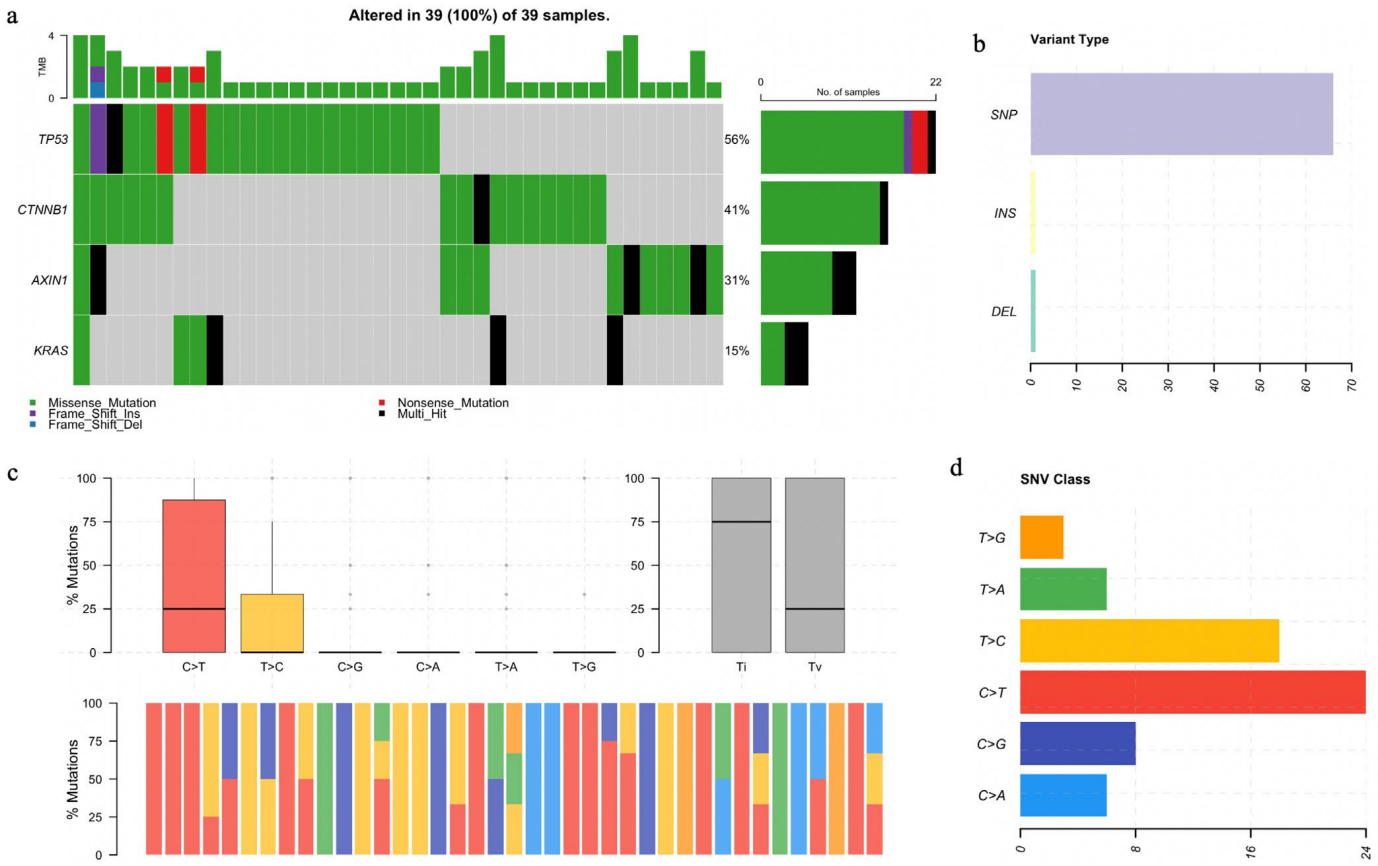
Mutational landscape of the HCC cohort. (a) The oncoplot displays the distribution and frequency of somatic mutations in *TP53*, *CTNNB1*, *AXIN1*, and *KRAS* across 39 liver tumor samples. Rows represent genes, and columns represent individual tumor samples. Mutation types are color‐coded as follows: Green (missense mutations), red (nonsense mutations), blue (frameshift deletions), purple (frameshift insertions), and black (multi‐hit mutations). The top bar plot shows the tumor mutation burden (TMB) per sample, and the right bar plot displays the mutation frequency for each gene. (b) Variant types in the HCC cohort are represented in different colors in the bar plots: single nucleotide variant (SNV), insertion (INS), and deletion (DEL). (c) Summary of SNV classifications and transition/transversion (Ti/Tv) ratios in the HCC cohort. (Top left): Bar plot showing the frequency of different single nucleotide substitutions (C>T, T>C, C>G, C>A, T>A, and T>G) in the cohort, with C>T transitions being the most prevalent, followed by T>C mutations. (Top right): Box plot summarizing the Ti/Tv mutation ratios, with transitions being more frequent than transversions. (Bottom panel): Stacked bar plots illustrating the proportional distribution of different substitution types across individual tumor samples. (d) Distribution of SNV classes in the HCC cohort.

We conducted a comprehensive mutational analysis of liver tumor samples from our HCC cohort, examining somatic mutations in MAF files using Maftools in R Studio. *JAK1* was excluded from further analysis as only SNPs were detected. Mutation frequencies and distributions for *TP53*, *CTNNB1*, *AXIN1*, and *KRAS* were summarized across 39 samples (Figure 4). *TP53* (56%), *CTNNB1* (41%), and *AXIN1* (31%) were the most frequently mutated genes, highlighting their potential involvement in tumorigenesis. *KRAS* mutations were observed in 15% of cases. Multi‐hit mutations were primarily observed in *TP53* and *AXIN1*, suggesting potential hotspot regions. The distribution of single nucleotide variants (SNVs) and their classifications was analyzed (Figure 4b). The most frequent substitution type was C>T, followed by T>C, with rarer substitutions (C>G, C>A, T>A, and T>G) observed less frequently (Figure 4c, top left). Transition/transversion (Ti/Tv) ratio analysis revealed that transitions predominated over transversions (Figure 4c, top right). The proportional distribution of SNVs across individual tumor samples showed variability, with C>T and T>C mutations being the most recurrent (Figure 4c, bottom panel). These findings suggest a mutational signature characteristic of HCC, potentially influenced by chronic inflammation or exposure to exogenous mutagens. This mutational landscape provides a clear visualization of the most frequently altered genes and the mutation distribution across the cohort, reinforcing the involvement of *TP53*, *CTNNB1*, and *AXIN1* in tumor progression.

### 3.4 Comparison With the TCGA Data Set and Statistical Analysis

**FIGURE 5:**
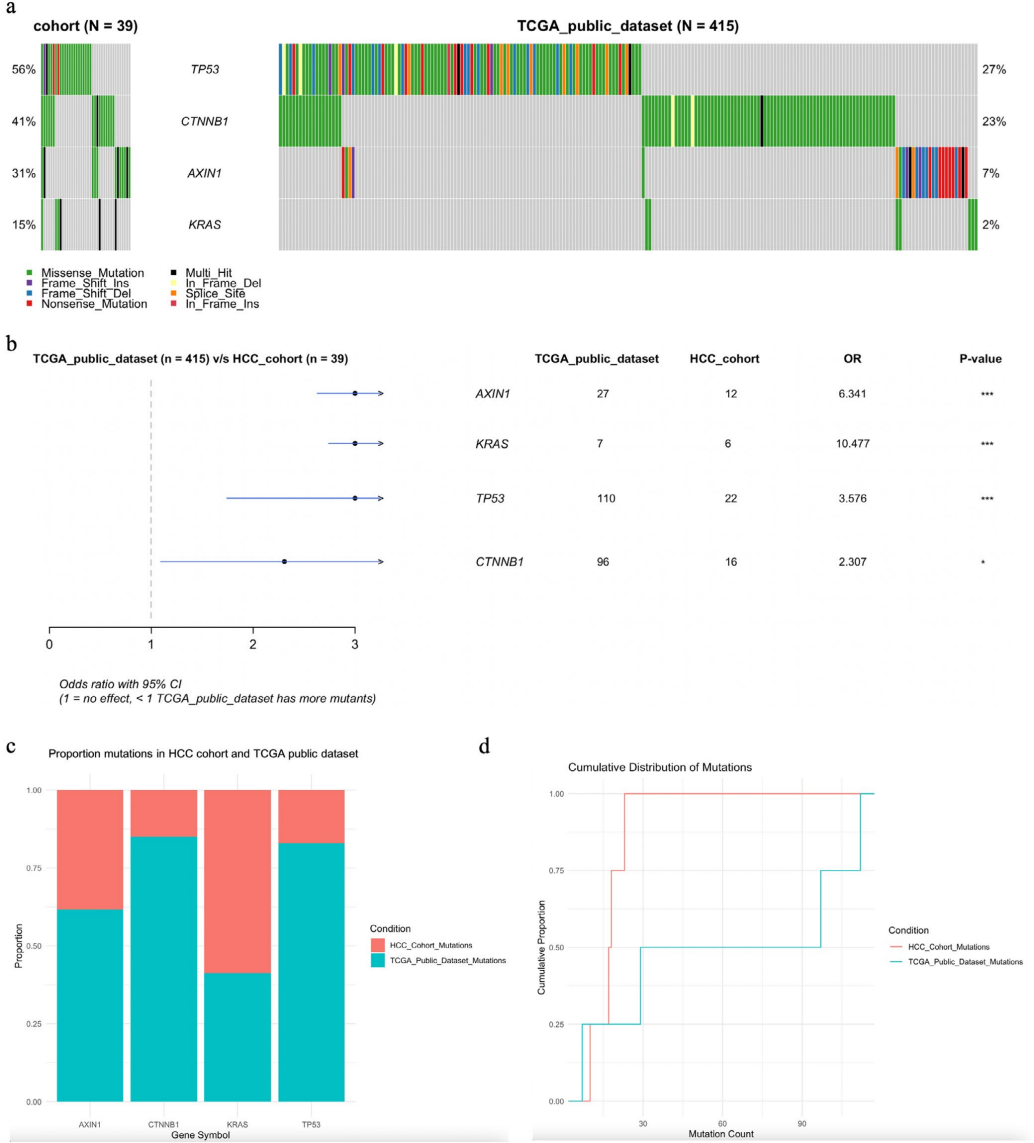
Comparison of mutations between our cohort and the TCGA data set. (a) Oncoplot showing the mutation landscape of key HCC‐associated genes (*TP53*, *CTNNB1*, *AXIN1*, and *KRAS*) in our cohort (*N* = 39, left) and the TCGA public data set (*N* = 415, right). Each column represents an individual sample, and different colors indicate various mutation types as described in the legend. The percentage of mutated cases for each gene is displayed on the left for our cohort and on the right for the TCGA data set. (b) Odds ratio analysis of key HCC‐associated genes in our cohort vs. the TCGA data set. Forest plot showing the odds ratio (OR) with 95% confidence intervals (CIs) for mutations in *AXIN1*, *KRAS*, *TP53*, and *CTNNB1* between our cohort (*N* = 39) and the TCGA data set (*N* = 415). OR values > 1 indicate a higher mutation frequency in our cohort compared to TCGA. Asterisks denote statistical significance (*p < 0.05, ***p < 0.001). Mutation counts for each gene in both data sets are provided in the table. (c) Proportion of mutations in the HCC cohort and TCGA data set. The stacked bar chart illustrates the proportion of mutated cases for *AXIN1*, *KRAS*, *TP53*, and *CTNNB1* in the HCC cohort (red) and the TCGA data set (blue). Each bar represents the total proportion of mutations for a given gene (scaled to 1), with the size of the red and blue portions reflecting the proportion of mutations in each data set, allowing a visual comparison of mutation distribution across cohorts. (d) Cumulative distribution of mutations across the HCC cohort and the TCGA data set. The red curve represents the HCC cohort, and the blue curve represents the TCGA data set. Differences in the shape and position of the curves highlight variations in mutation counts and the overall distribution of mutations between the two groups.

Comparing our cohort (*N* = 39) to the TCGA data set (*N* = 415), *TP53* and *CTNNB1* exhibited the highest mutation frequencies in both data sets, while *KRAS* had the lowest (Figure 5a). Despite its lower mutation frequency in our cohort, *KRAS* had a higher absolute mutation count compared to the TCGA data set (Figure 5b,c). The mutation distribution in our cohort was more concentrated, with multiple mutations detected within fewer samples, indicating a higher TMB (Figure 5d,e). In our cohort, *TP53* mutations were the most frequent (56%) compared to 27% in TCGA, while *CTNNB1* mutations were present in 41% of our cases, significantly higher than the 23% observed in TCGA. *AXIN1* mutations were found in 31% of our samples, whereas only 7% were reported in TCGA. *KRAS* mutations were present in 15% of our cohort, compared to 2% in TCGA. Missense mutations were predominant across all genes in both cohorts, but our cohort exhibited a higher proportion of frameshift deletions, nonsense mutations, and multi‐hit alterations, suggesting potential differences in mutational processes underlying HCC development in our population compared to the TCGA cohort.

An OR analysis was performed to compare the frequency of mutations in *AXIN1*, *KRAS*, *TP53*, and *CTNNB1* between our cohort and the TCGA data set (Figure 5b). *AXIN1* mutations were significantly more frequent in our cohort (OR = 6.341, *p* < 0.001), indicating a potential role in the molecular pathology of our patients. *KRAS* mutations were also highly enriched (OR = 10.477, *p* < 0.001), suggesting an alternative oncogenic pathway distinct from the TCGA cases. *TP53* mutations were more prevalent in our cohort (OR = 3.576, *p* < 0.001), emphasizing its critical role in tumorigenesis. In contrast, *CTNNB1* mutations were less significantly enriched (OR = 2.307, *p* < 0.05), suggesting possible differences in Wnt/β‐catenin signaling activation in our cohort compared to TCGA. These findings highlight distinct mutational patterns in our Mongolian HCC cohort, with a higher prevalence of *AXIN1*, *KRAS*, and *TP53* mutations, which may reflect unique genetic or environmental risk factors influencing liver carcinogenesis in this population.

We further compared mutation frequencies between our HCC cohort and the TCGA data set by analyzing the proportion of mutated cases in *AXIN1*, *KRAS*, *TP53*, and *CTNNB1* (Figure 5c). *AXIN1* and *KRAS* mutations represented a larger proportion of cases in our cohort, whereas *TP53* and *CTNNB1* mutations exhibited differing relative distributions across the data sets. This comparison offers an overview of mutation distribution, emphasizing cohort‐specific differences in the mutational landscape of HCC.

The cumulative distribution of mutations in our cohort was compared to the TCGA data set, showing a steeper rise in cumulative mutation proportions in our cohort (Figure 5d). This suggests that mutations are more frequent in our cohort, highlighting a higher mutation burden compared to the TCGA data set. The difference in the cumulative distribution between the two cohorts may reflect regional or etiological differences in mutation patterns.

### 3.5 Expression Levels of β‐Catenin and p53

**FIGURE 6:**
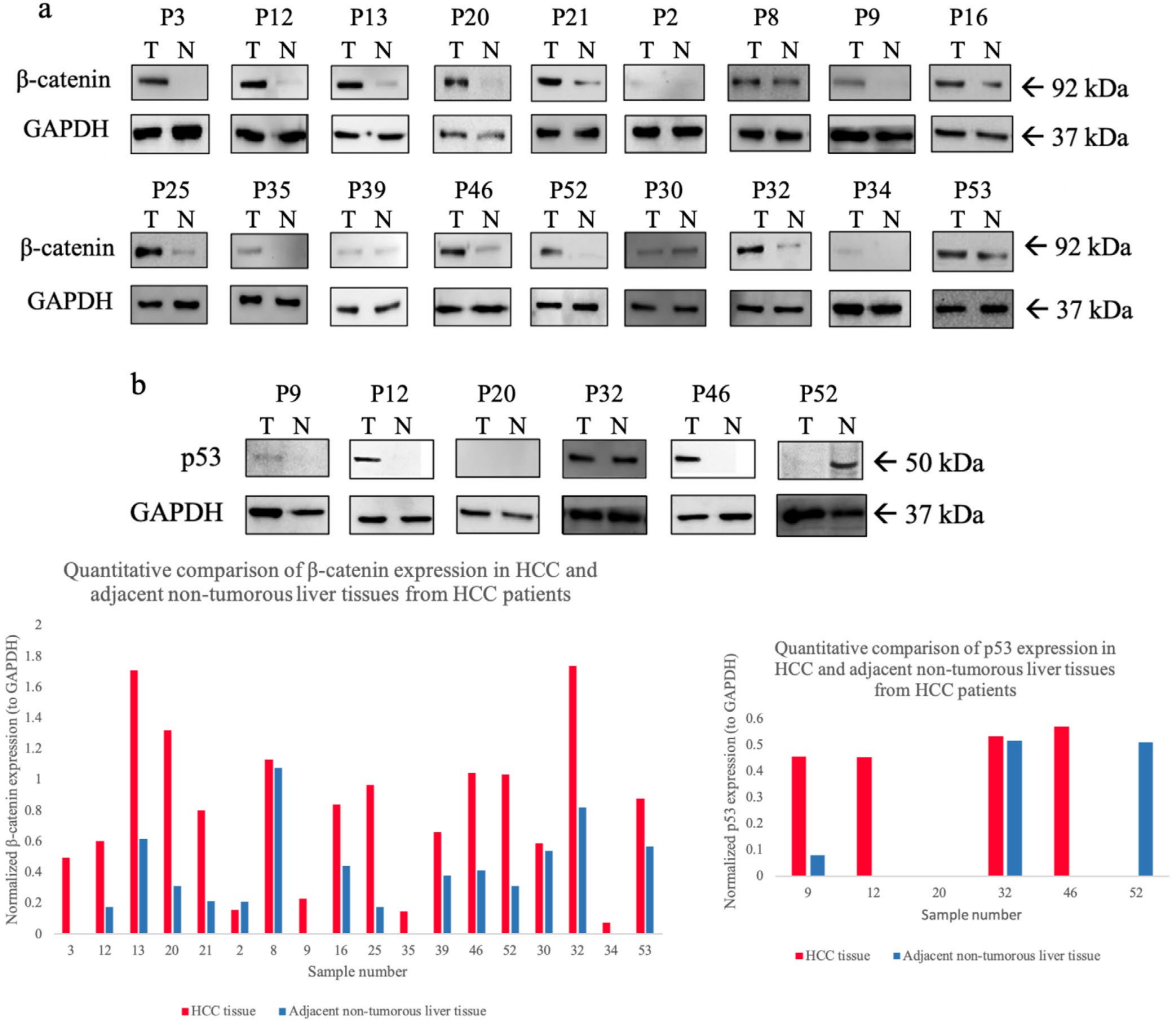
Western blot analysis of β‐catenin and p53 protein expression in HCC tumor and peritumoral tissue samples. GAPDH was used as a loading control for each sample. Tissue samples are labeled as P (Patient), with tumor tissue samples labeled as T (Tumor) and peritumoral tissue samples labeled as N (Normal). (a) Western blot probed with a monoclonal β‐catenin antibody. GAPDH was used as a loading control and detected with a monoclonal GAPDH antibody. HRP‐conjugated rabbit anti‐mouse antibody was used as the secondary antibody. The β‐catenin band was detected at ~92 kDa. (a′) Quantitative analysis of β‐catenin protein expression normalized to GAPDH, comparing HCC tumor tissues and peritumoral normal tissues. (b) Western blot probed with a monoclonal p53 antibody. GAPDH was used as a loading control. HRP‐conjugated rabbit anti‐mouse antibody was used as the secondary antibody. The p53 band was detected at ~50 kDa. (b′) Quantitative analysis of p53 protein expression normalized to GAPDH, comparing HCC tumor tissues and peritumoral normal tissues.

β‐Catenin expression was analyzed by Western blot in HCC tumor (*n* = 16) and peritumoral (*n* = 16) tissues. Elevated β‐catenin levels were observed in several tumor samples (T3, T12, T13, T16, T18, T20, T21, T25, T35, T46, and T52) compared to their corresponding peritumoral tissues, particularly in samples harboring *CTNNB1* mutations at phosphorylation sites (Figure 6a). Tumor samples T13, T35, and T46, carrying D32N and D32Y mutations, exhibited a 10.25‐fold increase in β‐catenin expression. Similarly, tumor samples T12 and T25, harboring the G34V mutation, showed a 15.25‐fold increase. Other *CTNNB1* mutations, including S37P, T41A, and S45P, were also associated with elevated β‐catenin expression. Notably, tumor samples with T41A mutations (T3, T20, and T21) exhibited 18.3‐ and 19.3‐fold increases in β‐catenin levels, respectively (Figure 6a). Overall, β‐catenin expression was elevated in 93.75% of HCC tumor samples with *CTNNB1* mutations, with an average increase of 11.21‐fold. Interestingly, in sample T9—despite the absence of C*TNNB1* mutations—β‐catenin levels were elevated by 16.4‐fold, suggesting the involvement of alternative regulatory mechanisms. Quantitative analysis of Western blot results confirmed that β‐catenin expression was significantly higher in HCC tumor tissues compared to peritumoral normal tissues (Figure 6a′; Supporting Information S2).

The expression of p53 protein in HCC tumor and peritumoral tissue samples (*n* = 12) was analyzed by Western blot. Increased p53 expression was observed in several tumor samples (T9, T12, T32, and T46) compared to their corresponding peritumoral tissues (Figure 6b). Variation in p53 protein levels was observed between tumor and peritumoral normal tissues, with the corresponding quantitative results shown in Figure 6b′ (Supporting Information S2). These findings support the differential expression patterns seen in the Western blot images. Among them, *TP53* mutations and polymorphisms were identified. For instance, the *TP53* F270I mutation was detected in T46, while no *TP53* mutation was found in T12. Additionally, T9 exhibited a slight increase in p53 expression compared to N9, where the S362S polymorphism was present. Conversely, p53 expression was absent in both tumor and peritumoral tissues of T20 due to a deletion in the 5′ spliceosome recognition sequence (–CAGGTCA–), which disrupted intron 7 splicing and led to a premature stop codon (TGA). Similarly, the *TP53* R213* mutation in T52 resulted in a truncated p53 protein, leading to loss of p53 expression in the tumor. Interestingly, in peritumoral tissue N52, p53 was still overexpressed. These findings indicate that *TP53* mutations contribute to either increased or abolished p53 expression in HCC tissues.

Except for T16, all HCC patients harboring *CTNNB1* and *TP53* mutations were infected with hepatitis viruses. Specifically, cases T3, T8, T12, T13, T32, and T46 were infected with HCV, while T20 and T25 had HBV infections. Case T21 was co‐infected with HCV and HBV, case T35 carried HBV and HDV, and case T52 was infected with HCV, HBV, and HDV.

### 3.6 *CCND1* Gene Expression

**FIGURE 7:**
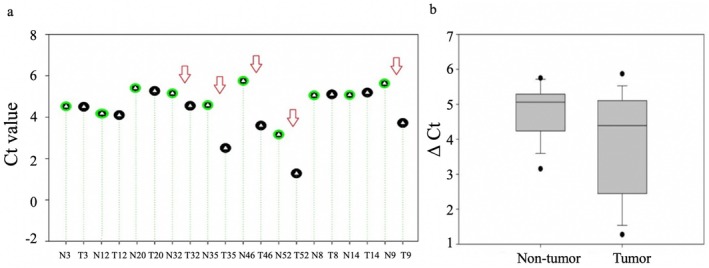
*CCND1* gene expression in HCC tumor and peritumoral tissue samples. (a) *CCND1* expression levels in HCC tumor and peritumoral tissue samples, represented by individual Ct values. (b) Comparison of *CCND1* expression between HCC tumor and peritumoral tissue samples based on average Ct values.

The expression of *CCND1*, a downstream target gene of β‐catenin in the canonical Wnt/β‐catenin signaling pathway, was analyzed by qPCR in HCC (*n* = 10) and peritumoral tissue samples (*n* = 10) with *CTNNB*1 gene alterations. Elevated *CCND1* expression was observed in tumor tissues (T1, T32, T35, T46, and T52) compared to their corresponding peritumoral tissues (*p* = 0.02213) (Figure 7a), as illustrated in the box plot (Figure 7b). The average Ct value for *CCND1* expression was lower in tumor tissues (4.42) compared to peritumoral tissues (5.05), corresponding to a higher gene expression in the tumor samples.

Tumor samples T32 and T52, which contained *CTNNB1* mutations (S45P and S37P), showed elevated *CCND1* expression at the critical phosphorylation sites of β‐catenin, which were consistent with increased β‐catenin protein levels in these tumor tissues. No significant difference in *CCND1* expression was found between tumor and peritumoral tissues in samples T1, T13, and T22, despite β‐catenin overexpression in the tumors. The correlation between β‐catenin protein levels and *CCND1* expression was moderate (*r* = 0.42703), indicating a potential link between β‐catenin activation and *CCND1* expression in these HCC samples.

## 4 Discussion

In this study, *TP53* mutations were the most prevalent, occurring in 41.81% of Mongolian HCC patients. A significant proportion (84%) of these mutations were missense variants within the DNA‐binding domain, likely impairing p53's ability to bind DNA and recognize DNA damage [30]. Within this domain, the F270 residue plays a crucial role in stabilizing p53 and facilitating DNA interaction [31]. In HCC tissues harboring the F270I mutation, elevated p53 levels were observed, suggesting a structural disruption that weakens DNA binding, leading to p53 accumulation and potentially contributing to tumor progression. Additionally, a moderate increase in p53 expression was noted in samples with the S362S polymorphism. While phosphorylation at S362 by Inhibitory Kappa B kinase beta (IKK2) is known to stabilize p53 [32], the S362S polymorphism itself is not directly linked to p53 stabilization, indicating that alternative regulatory mechanisms may influence p53 expression. Interestingly, p53 expression was absent in the HCC tissue of sample T52 with an R213 mutation, which introduces a stop codon. The R213 residue is critical for p53 stability, its interaction with the DNA backbone, and essential co‐factors [33]. The R213 mutation truncates p53 translation, leading to the loss of its tumor suppressor function and likely contributing to tumor development in this sample. Similarly, the deletion of a 5′ spliceosome recognition sequence (–CAGGTCA–) in the HCC sample (T20) caused the retention of intron 7, which contained a stop codon (TGA) and resulted in p53 translation termination. This mutation mirrors findings by Lai et al. [34], who reported similar inactivation of p53 due to a stop codon in intron 7. The *TP53* R249S mutation is one of the most frequently observed mutations in HCC, particularly in regions with high aflatoxin B1 exposure [35, 36, 37], such as Asia and sub‐Saharan Africa. However, this mutation was absent in our cohort, suggesting a lower prevalence of aflatoxin exposure in Mongolian HCC patients. Instead, hepatitis virus infections were more strongly associated with HCC in our study population, indicating that viral infections may represent the primary etiological factor.

Missense mutations in exon 3 of the *CTNNB1* gene were identified in 29.09% of HCC samples, leading to amino acid substitutions at D32, S33, G34, S37, T41, and S45—residues commonly mutated in HCC. Notably, tumors harboring D32N/Y and G34V mutations exhibited 10.25‐fold and 15.25‐fold increases in β‐catenin accumulation compared to their corresponding peritumoral tissues. While D32 and G34 residues are not directly phosphorylated, they are essential for facilitating β‐catenin phosphorylation by the F‐box/WD repeat‐containing protein 1A (FBXW1A) ubiquitin ligase [38]. Mutations at these sites likely disrupt phosphorylation, resulting in β‐catenin stabilization and activation of the Wnt/β‐catenin signaling pathway. Similarly, tumors harboring S33, S37, and T41 mutations exhibited significantly elevated β‐catenin levels. These residues, located within the N‐terminal destruction box, are normally phosphorylated by glycogen synthase kinase‐3β (GSK‐3β), marking β‐catenin for degradation [38]. Mutations at these positions likely impair degradation, leading to β‐catenin accumulation in HCC samples. Additionally, in tumors with the S45P mutation, β‐catenin levels were elevated 8.4‐fold relative to peritumoral tissue. Since phosphorylation of S45 by casein kinase 1 alpha (CK1α) initiates subsequent phosphorylation events, mutations at this site may promote β‐catenin stabilization and oncogenic activation [39].

To further investigate the functional impact of these mutations, the expression of *CCND1*, a downstream target of the Wnt/β‐catenin pathway, was analyzed in HCC tissues with elevated β‐catenin levels. Overexpression of *CCND1* was observed in samples harboring *AXIN1* S428S and *CTNNB1* S45P, D32Y, and S37P mutations, each associated with different hepatitis virus infections (HBV, HCV, and HDV). These findings suggest that viral infections may contribute to the activation of the Wnt/β‐catenin pathway in HCC. Furthermore, a significant correlation was observed between *CTNNB1* mutations and hepatitis virus infections (*p* < 0.01), reinforcing the potential role of viral infections in Wnt/β‐catenin pathway activation.

β‐Catenin is integrated with *AXIN1* in the destruction complex through phosphorylation at Ser33, Ser37, Ser45, and Thr41, as reported by Tornesello et al. [40]. Their study also identified *CTNNB1* mutations at serine and threonine residues in HCC patients with HCV, accounting for 17.5%, which is slightly lower than the proportion observed in our study. Similarly, Rebouissou et al. [41] reported that oncogenic mutations at positions D32 and S37 were highly prevalent (56%), whereas mutations at S45 and T41 were detected at lower frequencies (27% and 14%, respectively) in HCC patients. Furthermore, *CTNNB1* mutations were frequently observed in HCC patients with hepatitis [42], indicating their potential relevance. In the present study, 91.6% of HCC patients with *CTNNB1* mutations were diagnosed with hepatitis viral infections, further supporting this association. Wang et al. [43] reported that *AXIN1* mutations contribute to the inactivation of the β‐catenin destruction complex, accounting for 8%–10% of alterations in the Wnt/β‐catenin signaling pathway in HCC. These mutations typically occur in both the β‐catenin binding domain and the GSK3β/CK1α binding sites [44], potentially disrupting the regulatory function of *AXIN1*. In our study, *AXIN1* mutations were identified in 21.8% of HCC cases, a higher prevalence than previously reported. These mutations were primarily located in the β‐catenin binding domain, suggesting a potential disruption in β‐catenin scaffolding that may impair its interactions within the Wnt/β‐catenin signaling pathway. Additionally, *AXIN1* polymorphisms were detected in 10.9% of HCC patients, further supporting the role of *AXIN1* genetic alterations in tumor progression. Our findings align with those of Gao et al. [38], who also reported that β‐catenin accumulation can occur independently of *CTNNB1* mutations, driven instead by *AXIN1* mutations. This suggests that β‐catenin regulation is not solely dependent on *CTNNB1* status but is influenced by the overall integrity of the destruction complex.

*KRAS* oncogenic mutations account for approximately 25% of all human cancers [45]. Notably, Hou et al. [46] reported that cancers most strongly associated with *KRAS* mutations include pancreatic cancer (80%), cholangiocarcinoma (45%), and lung cancer (32%), all of which exhibit higher mutation frequencies than HCC, where the *KRAS* mutation rate is approximately 5.6%. Other studies have reported a similar mutation rate of around 7% in liver cancer [47], suggesting that *KRAS* mutations are typically present in fewer than 10% of HCC cases. However, our study identified *KRAS* mutations in 10.9% of the HCC cohort, a relatively higher mutation rate compared to previous reports. Notably, while the G12C substitution is the most common *KRAS* mutation in Chinese populations [48], it was absent in our cohort. Instead, the G60V substitution, which accounted for 33.3% of *KRAS* mutations in Mongolian HCC patients in our study, was notably more prevalent compared to only 0.2% in Chinese populations. This divergence underscores the genetic diversity of *KRAS* mutations across different populations and suggests the potential for population‐specific therapeutic strategies or genetic screening methods.

In this study, three common polymorphisms in the *JAK1* gene were identified: A699A at 8%, and S683S and P733P at 24% and 68%, respectively. Compared to head and neck squamous carcinoma, where these polymorphisms are found at frequencies of 5%, 30%, and 25%, respectively [49], the frequency of P733P in our cohort was 2.7 times lower. Additionally, two common polymorphisms (193 and 1032) described by De Carvalho et al. [49] were not identified in our cases, suggesting organ‐specific variations. Although amino acid substitution was not found in this study, Xie et al. [28] identified silent and missense mutations in HCC, which may provide further cancer growth in humans.

Numerous studies have explored the association between hepatitis virus infections and liver cancer globally [6, 46]. In our cohort, HCV mono‐infection was notably prevalent (81.8%) among HCC cases, a significantly higher rate compared to the 53.2% reported by Oyunsuren et al. [50]. Consistent with previous studies, HCV infection has been shown to inhibit p53 activation, independent of Mouse Double Minute 2 (MDM2) or proteasomal degradation [51]. Additionally, the hepatitis B virus X (HBx) protein has been demonstrated to act as a transcriptional repressor by binding to the *TP53* promoter, thereby suppressing *TP53* expression in HBV‐related HCC [52]. In our study, 73.91% of *TP53* mutations were found in cases with hepatitis infections, further supporting the role of viral infections in *TP53* alteration. HBx also plays a direct role in activating the Wnt/β‐catenin signaling pathway, inhibiting β‐catenin degradation and promoting its accumulation [40]. In line with this, 84% of *CTNNB1* mutations in our study were detected in hepatitis‐infected HCC cases. Li et al. [12] reported that wild‐type *AXIN1* suppresses tumor progression, while mutant *AXIN1* in HBV‐related HCC not only loses this suppressive function but also promotes tumorigenesis. Similarly, 83.33% of *AXIN1* mutations in our cohort were identified in hepatitis‐infected cases, suggesting a potential link between these mutations and viral infections. While not all *JAK1* polymorphisms are associated with HBV infection, some have been linked to its effects [24]. In our study, all detected *JAK1* alterations were classified as polymorphisms (45.45%), and 76% of these polymorphisms co‐occurred with mutations in other key genes, including *TP53*, *CTNNB1*, *AXIN1*, and *KRAS*. This co‐occurrence suggests that *JAK1* polymorphisms may contribute to HCC pathogenesis, particularly in the context of viral infections.

In this study, we conducted a comprehensive mutational analysis of HCC samples from a cohort of 39 patients with mutations, focusing on key driver genes such as *TP53*, *CTNNB1*, *AXIN1*, and *KRAS*. Our findings revealed a significantly higher mutation frequency in *TP53*, *CTNNB1*, and *AXIN1* in our cohort compared to the TCGA data set, suggesting the potential involvement of these genes in HCC pathogenesis within the Mongolian population. Notably, *AXIN1* and *KRAS* mutations were highly enriched in our cohort, with odds ratios indicating a distinct molecular profile in comparison to TCGA cases. This suggests that the molecular mechanisms driving HCC in our cohort may differ, potentially influenced by unique genetic or environmental factors prevalent in this population.

The higher mutation burden observed in our cohort, characterized by a concentration of multi‐hit mutations, especially in *TP53* and *AXIN1*, underscores the importance of these genes in the molecular pathogenesis of HCC. Our findings also align with the established role of *TP53* in liver carcinogenesis, as mutations in this tumor suppressor gene are commonly associated with various malignancies. The enriched mutations in *KRAS* and *AXIN1*, however, point to the potential for alternative oncogenic pathways being involved, suggesting a more complex etiology of HCC in this population.

Additionally, the transition mutations (C>T and T>C) observed across both our cohort and TCGA data set may be indicative of a mutational signature common to HCC, possibly influenced by chronic liver diseases, inflammation, or external mutagens. These findings provide a clearer understanding of the mutational landscape of HCC in the Mongolian population and contribute to the broader understanding of HCC genetics globally.

Overall, our study underscores the complex interplay between genetic alterations and viral infections in HCC pathogenesis, highlighting the need for targeted therapeutic interventions and genetic screening methods in affected populations.

## 5 Conclusion

In this study, we identified somatic mutations and polymorphisms in several oncogenic and tumor suppressor genes associated with HCC. Notably, *TP53* genetic alterations, including F270I and S362S, led to increased p53 protein levels in HCC tumor tissues. In contrast, R213 and a short sequence deletion (–CAGGTCA–) upstream of intron 7 introduced premature stop codons, leading to truncated p53 proteins with impaired tumor suppressor function, potentially contributing to tumor progression in Mongolian HCC patients. All *TP53*‐altered cases were diagnosed with hepatitis virus infections, highlighting a potential link between viral hepatitis and *TP53*‐driven tumorigenesis.

In addition, *CTNNB1* mutations (D32N/Y, S33C/Y, S34V, S37P, T41A, and S45P) were detected in 93.75% of hepatitis‐related HCC cases, showing a strong correlation between these mutations and hepatitis virus‐associated HCC. These mutations, occurring at phosphorylation sites within the β‐catenin destruction box, disrupted GSK‐3β and CK1α‐mediated phosphorylation, preventing proteasomal degradation and leading to β‐catenin accumulation. Consequently, *CCND1* gene expression, a key downstream target of β‐catenin, was significantly upregulated in tumor tissues compared to peritumoral tissues (*p* = 0.02213), and its expression correlated positively with β‐catenin accumulation (*r* = 0.42703). This supports the oncogenic activation of the Wnt/β‐catenin signaling pathway in hepatitis‐associated HCC in Mongolian patients.

In our study, *AXIN1* mutations were found to co‐occur with *CTNNB1* mutations in 37.5% of HCC cases, highlighting their frequent association. Notably, the *AXIN1* S428 alteration specifically contributed to increased β‐catenin levels, further supporting its role in β‐catenin accumulation.

Regarding *KRAS* mutations, half were located within switch residues of the catalytic subdomain, potentially inducing conformational changes that disrupt K‐Ras GTPase activity. Notably, the G60V substitution accounted for 33% of all *KRAS* mutations, highlighting its potential oncogenic role. The remaining mutations were found in the allosteric region of the G domain, which may impact enzymatic function and downstream signaling activation, potentially driving HCC development. Among all *KRAS* mutations detected in this study, half were located in the switch residues of the catalytic subdomain, suggesting conformational changes that could impair the GTPase activity of the K‐Ras protein. The G60V substitution constituted 33% of all *KRAS* mutations in HCC cases, suggesting its potential oncogenic significance. The other half of the *KRAS* mutations were identified in the allosteric region of the G domain, a critical site for enzymatic regulation. Given the functional significance of this region, these mutations could alter enzymatic activity and disrupt downstream signaling, potentially contributing to cancer development in Mongolian HCC patients.

Although all genetic alterations in *JAK1* identified in this study were classified as polymorphisms without amino acid changes in HCC cases, their frequency was higher than that of mutations detected in the other four genes analyzed (*TP53*, *CTNNB1*, *KRAS*, and *AXIN1*). Notably, 76% of cases with *JAK1* polymorphisms co‐occurred with mutations in these other genes, suggesting a potential correlation between *JAK1* polymorphisms and these mutations. Furthermore, 80% of *JAK1* polymorphisms were found in hepatitis‐related HCC cases, indicating that hepatitis infection may contribute to the occurrence of *JAK1* polymorphisms. Together, these findings suggest that *JAK1* polymorphisms may be linked to both key genetic mutations and hepatitis‐related HCC.

In conclusion, our study provides valuable insights into the mutational landscape of HCC in the Mongolian population, highlighting significant differences in mutation frequencies, especially in *TP53*, *CTNNB1*, *AXIN1*, and *KRAS*. The higher mutation burden observed in our cohort, alongside the enrichment of *AXIN1* and *KRAS* mutations, suggests the presence of distinct oncogenic pathways in the development of HCC, which may be influenced by regional genetic and environmental factors. These findings emphasize the need for further research into the specific genetic drivers of HCC in different populations, which could have important implications for the development of targeted therapies and personalized treatment strategies in liver cancer. Ultimately, 85.4% of HCC patients in this study were diagnosed with hepatitis virus infections, and mutations in oncogenes and tumor suppressor genes were significantly associated with viral infections and specific viral subtypes (*p* < 0.01). These findings provide insights into the molecular mechanisms driving HCC pathogenesis, highlighting the potential interplay between viral hepatitis and key oncogenic pathways. Further studies are warranted to investigate the clinical implications of these genetic alterations and their potential as therapeutic targets for HCC.

## Author Contributions

G.D. conceived the research of the presented idea and planned the experimental design. An.E., G.E.B., and T.N. collected biopsies from patients diagnosed with HCC. N.M., Z.S., and D.D. contributed to sample preparation and RNA isolation. K.B. carried out cDNA synthesis. Ar.E. and N.O. contributed to DNA isolation. For the PCR performance, K.B. (TP53 and JAK1), Ar.E. and G.B. (CTNNB1 and AXIN1), and N.M. and Z.S. (KRAS) contributed. N.B. and D.D. contributed to western blotting and qPCR experiments. K.B. and Ar.E. acquired the data and interpreted it, and wrote the PCR and western blot results. D.D. wrote the Materials and Methods. N.B. wrote the manuscript, designed graphics, analyzed the graphical data, and concluded the results and discussion. T.O. and N.B. revised the manuscript. T.B. organized documentation and ethical approval for the research. T.O. supervised the project.

## Ethics Statement

The present study was approved by the Ethics Committee of the Ministry of Health of Mongolia on June 3, 2021 (Ethical approval no. 220) (National Registration No. 202300080).

## Consent

Informed consent was obtained from all individual participants included in the study.

## Conflicts of Interest

The authors declare no conflicts of interest.

## Supporting information


**Data S1.** Supporting Information.

**Data S2.** Supporting Information.

## Data Availability

Data are available in Supporting Information S2.
