# Supplementary material for: High‐Frequency Mutations in TP53 , AXIN1 , CTNNB1 , and KRAS , and Polymorphisms in JAK1 Genes Among Mongolian HCC Patients
Source: Cancer Rep (Hoboken). 2025 May 8;8(5):e70227. doi: 10.1002/cnr2.70227 (PMC12062512; doi:10.1002/cnr2.70227)
Supplement: Supplementary file 1 — Data S1. Supporting Information. [file CNR2-8-e70227-s002.docx]

Supporting Information 1

DNA sequencing results of *CTNNB1,* *TP53*, *KRAS*, *AXIN1* and *JAK1* genes in the HCC patients are shown Figure 1-5.


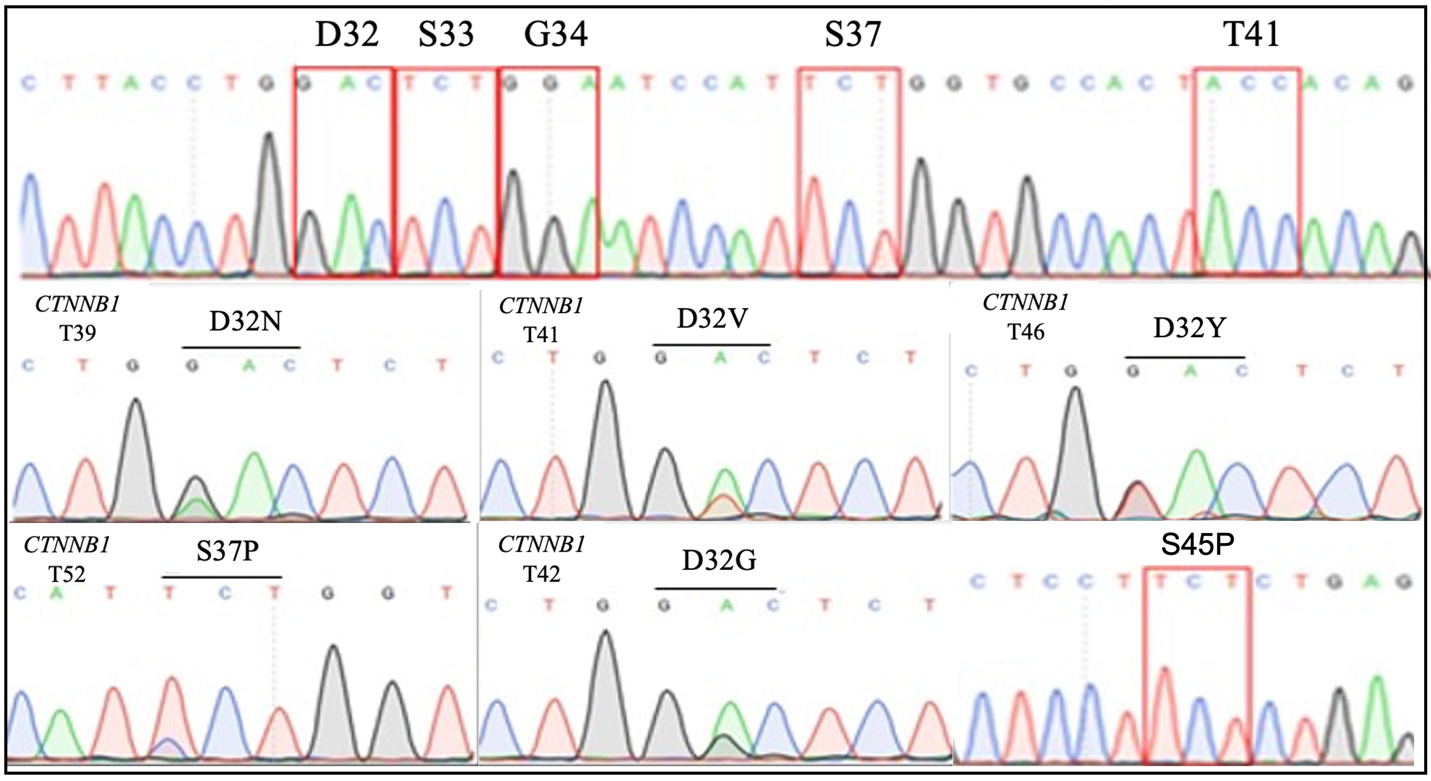


Figure 1. *CTNNB1* gene sequencing in the HCC samples. *CTNNB1* mutations are labeled on the figure.


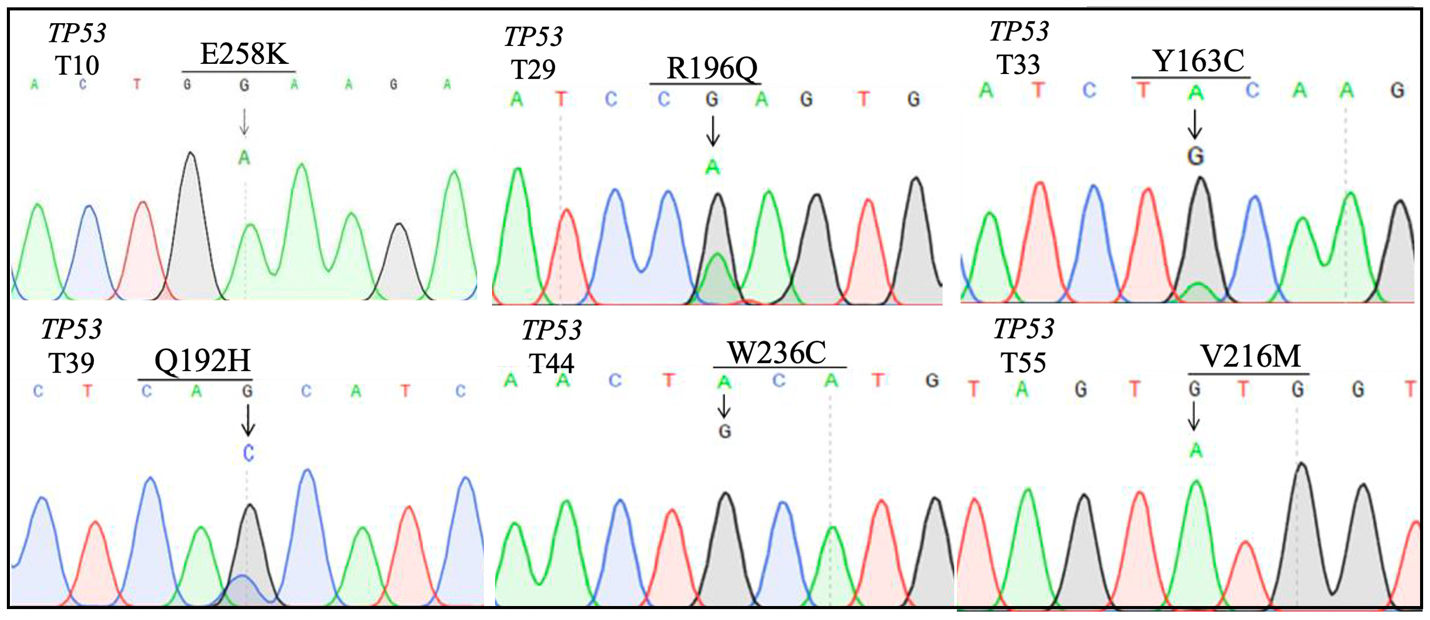


Figure 2. *TP53* gene sequencing in the HCC samples. *TP53* mutations are labeled on the figure.


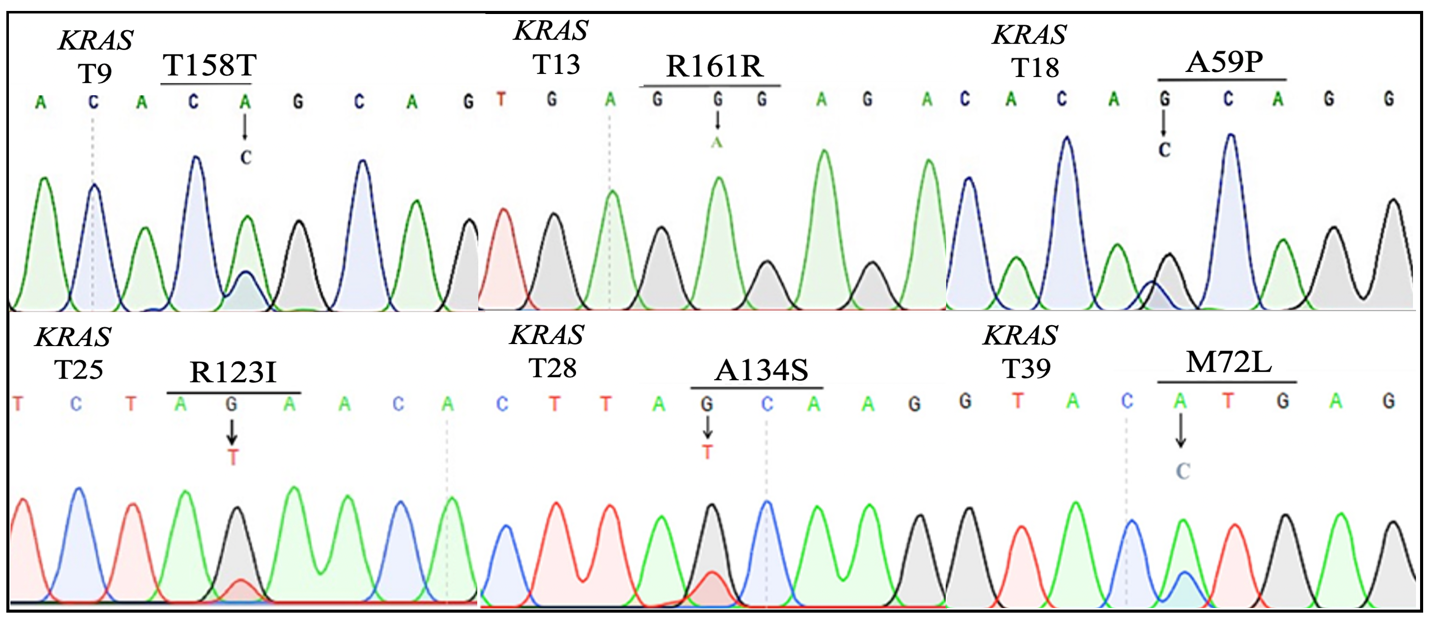


Figure 3. *KRAS* gene sequencing in the HCC samples. *KRAS* mutations are labeled on the figure.


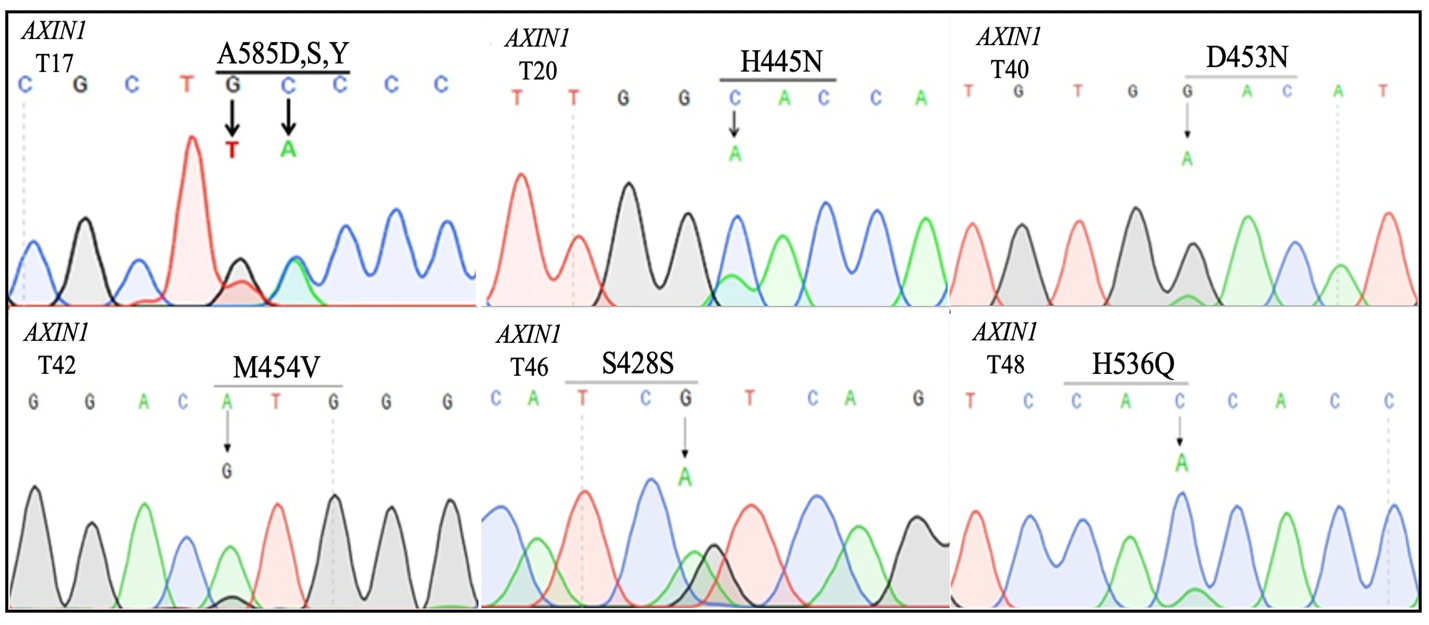


Figure 4. *AXIN1* gene sequencing in the HCC samples. *AXIN1* mutations are labeled on the figure.


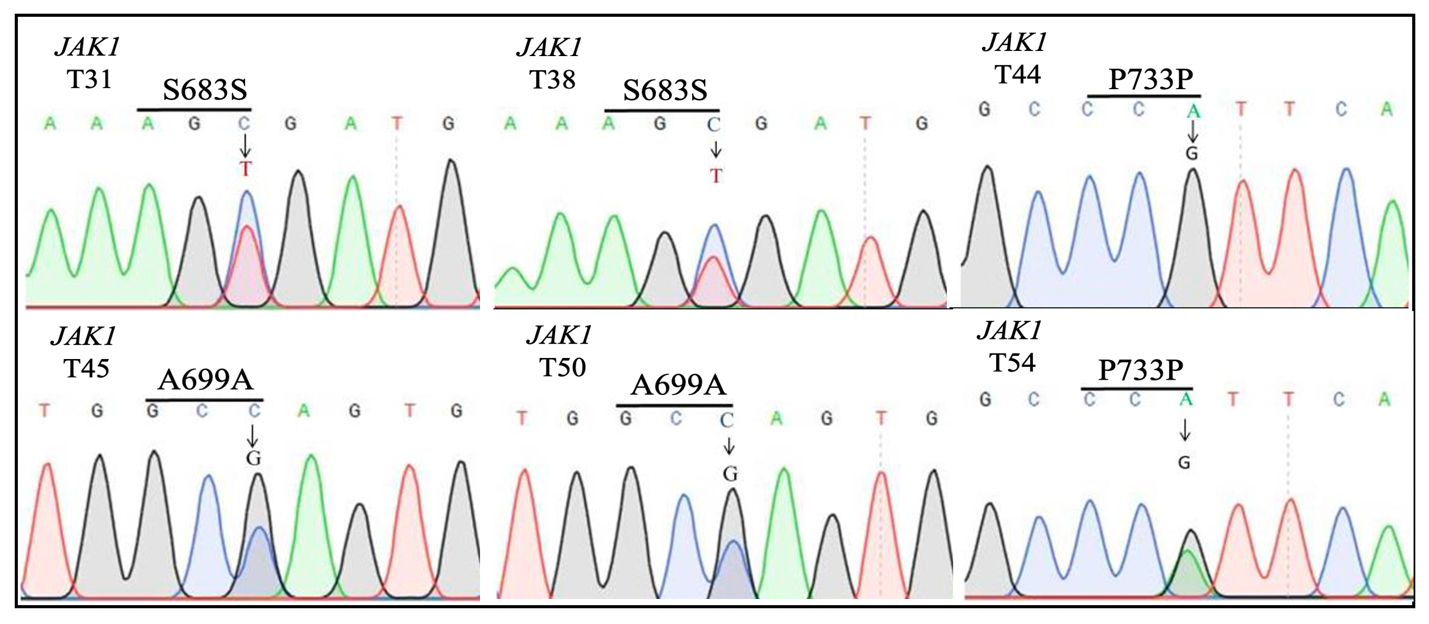


Figure 5. *JAK1* gene sequencing in the HCC samples. *JAK1* mutations are labeled on the figure.
